# Asymptomatic Tattoo-Associated Cutaneous Nontuberculous Mycobacterial Infection: Case Report

**DOI:** 10.2196/108587

**Published:** 2026-10-30

**Authors:** Chris Alihosseini, Milana Stein, Ana Sofia Acosta Madiedo, Roksana Hesari, Matthew Kauffman, Martin N Zaiac

**Affiliations:** 1 Department of Dermatology, Larkin Community Hospital, Hialeah, FL, United States; 2 HCA Florida Westside Hospital, 8201 W Broward Blvd, Plantation, FL, 33324, United States, 1 9175445782; 3 Florida International University Herbert Wertheim College of Medicine, Miami, FL, United States; 4 Department of Dermatology, Herbert Wertheim College of Medicine, Miami, FL, United States

**Keywords:** dermis, epidermis, microbiome, mycobacteria, tattoo-associated infection

## Abstract

Tattoo-associated nontuberculous mycobacterial infections can present in a variety of ways, including papular, pustular, nodular, and abscess-like morphologies. We report the case of an immunocompetent 28-year-old male who presented with diffusely distributed monomorphic papules with collarettes of scale confined to recently tattooed skin. Histopathologic evaluation demonstrated an atypical lichenoid vacuolar interface dermatitis, and tissue culture ultimately confirmed infection with *Mycobacterium abscessus* complex following prolonged incubation. This case highlights the diagnostic challenges posed by atypical clinical and histopathologic features of tattoo-associated nontuberculous mycobacterial infections, emphasizing the importance of early biopsy, mycobacterial culture, and prolonged combination antimicrobial therapy for optimal patient outcomes.

## Introduction

Tattoo-associated cutaneous reactions are increasingly encountered in dermatologic practice and are most commonly inflammatory or allergic in nature [1]. However, infectious complications must be considered in atypical cases. Nontuberculous mycobacteria (NTM) are environmental organisms capable of causing skin and soft tissue infections through direct inoculation, including during tattooing. Rapidly growing mycobacteria, such as *Mycobacterium abscessus (M abscessus)*, are being frequently implicated in tattoo-related cases. A 2026 systematic review identified 311 individuals across 15 countries with tattoo-associated NTM infection, with 29 of the 67 infection events being in the United States [2]. More specifically, in a 2015 Miami-Dade County outbreak, 38 of 226 interviewed tattoo clients were identified as outbreak-associated cases of *M abscessus* from contaminated graywash ink and tap water [3]. Exposure to gray tattoo ink was associated with an increased likelihood of developing a rash. These infections may be underrecognized due to their indolent course, variable morphology, and delayed culture growth. Awareness of tattoo-associated NTM infections is critical for timely diagnosis and appropriate management, as misdiagnosis can result in delayed appropriate therapy, unnecessary treatments, and prolonged morbidity [4]. We present a case of tattoo-associated cutaneous NTM infection in a young immunocompetent male, highlighting diagnostic challenges, atypical histopathologic findings, and important management considerations for dermatologists.

## Case Report

A 28-year-old male presented with an asymptomatic rash involving tattooed skin on the right upper arm and left calf 2 months after the initial tattoo session. The patient denied pain, pruritus, fever, chills, lymphadenopathy, drainage, or other systemic symptoms. He had no significant past medical history, was not immunocompromised, and reported no recent illness. The absence of constitutional or local symptoms, despite involvement of two anatomically distinct tattoo sites, made the presentation atypical for a clinically apparent infection and contributed to diagnostic complexity.

Physical examination demonstrated diffusely distributed monomorphic papules confined predominantly to areas of gray and blue tattooed skin, as well as some small papules with no erythema and with collarettes of white scale (Figure 1). The multifocal involvement of distinct tattoo sites raised suspicion for an inoculation-related etiology rather than a localized allergic or inflammatory reaction. Given the chronicity, morphology, and confinement to tattoo pigment, an infectious process was included early in the differential diagnosis, despite the lack of classic infectious symptoms.

**Figure 1.**
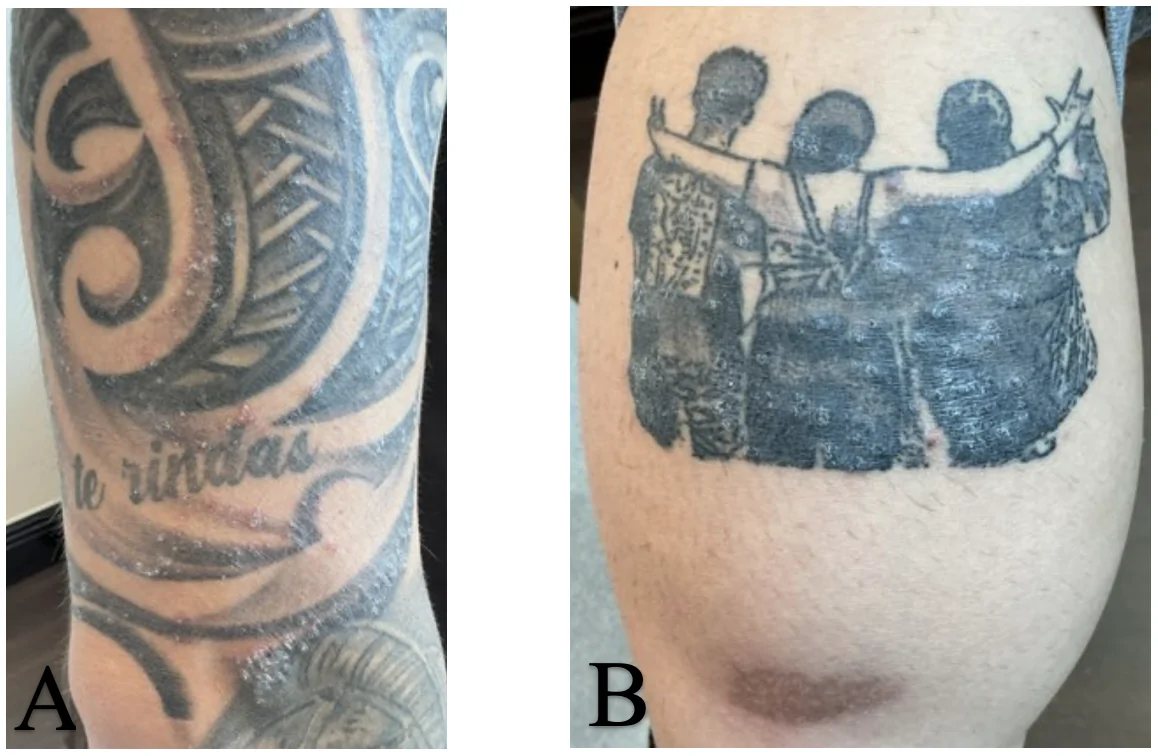
(A) Right upper arm showing diffusely distributed monomorphic papules confined predominantly to areas of gray and blue tattoo pigment and (B) left calf with evidence of small papules with and without surrounding erythema, some exhibiting collarettes of white scale consistent with multifocal tattoo-associated cutaneous involvement.

A punch biopsy (Figure 2) was obtained from the right upper arm for histopathologic evaluation and tissue culture, including an acid-fast bacilli culture. Histopathology revealed acute folliculitis with a lichenoid vacuolar lymphohistiocytic interface dermatitis associated with tattoo pigment (Figure 3). Although granulomatous inflammation is frequently described in cutaneous NTM infection, the presence of a lichenoid vacuolar interface pattern in this case was atypical and could have supported an inflammatory or lichenoid tattoo reaction in the absence of microbiologic testing.

Initial empiric treatment consisted of twice-daily triamcinolone acetonide 0.1% ointment and doxycycline 100 mg for one week, reflecting standard management for inflammatory tattoo reactions while awaiting culture results. The acid-fast bacilli culture subsequently isolated a NTM species, confirming the diagnosis of tattoo-associated cutaneous NTM infection. The prolonged incubation period required for organism growth demonstrated a key diagnostic delay inherent to these infections.

Following microbiological confirmation of *M abscessus* complex, the treatment regimen was escalated. Prolonged dual antibiotic therapy was initiated with twice-daily doxycycline 100 mg and clarithromycin 500 mg for a planned duration of 3 months as per current recommendations on length of therapy for mild skin and soft tissue infections by NTM and dual-agent therapy to minimize the risk of antimicrobial resistance [5,6].

*M abscessus* complex is a group of NTM, characterized by fast-growing, nonchromogenic properties, and it is well known for its multidrug resistance [7]. For example, *M abscessus* complex accounted for 12 of 67 reported infection events in Miami in 2015, making it the second most frequently identified species after *Mycobacterium chelonae* [2].

**Figure 2.**
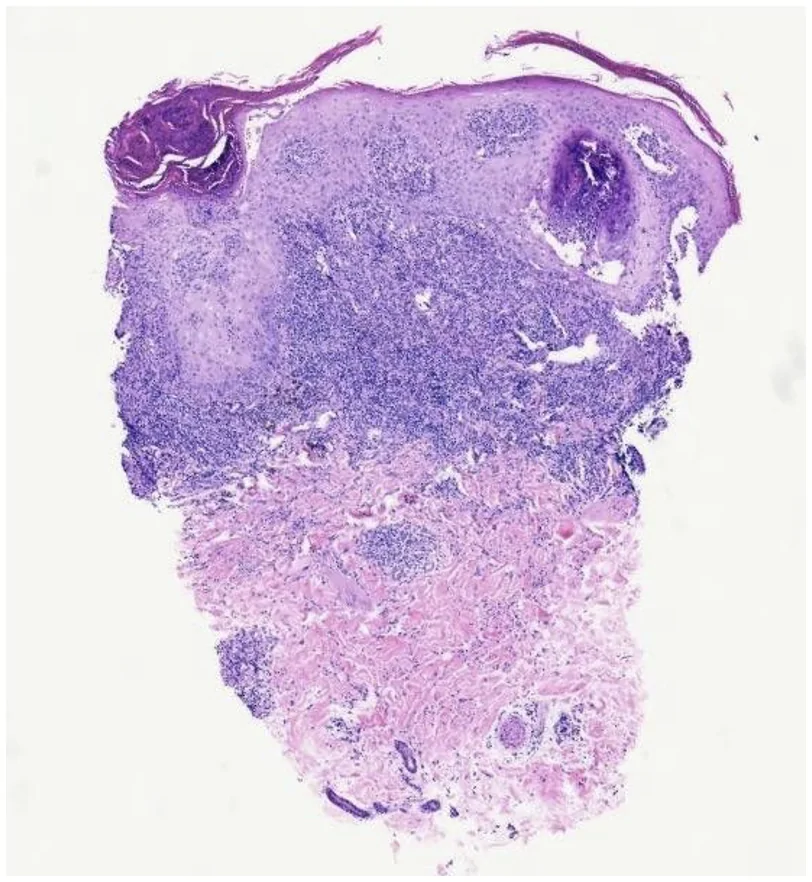
Punch biopsy from patient’s right upper arm.

**Figure 3.**
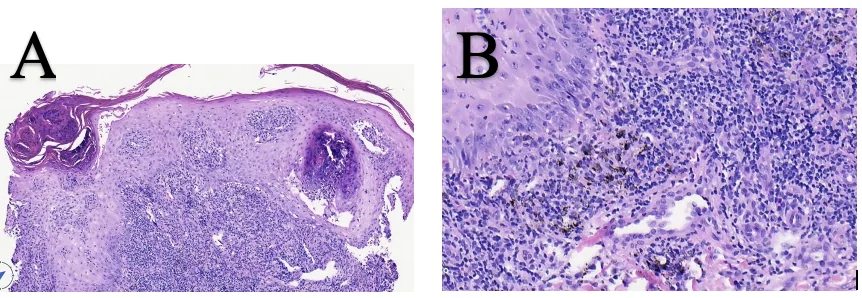
(A) Histopathology of punch biopsy demonstrating acute folliculitis with lichenoid vacuolar lymphohistiocytic interface dermatitis associated with tattoo pigment; hematoxylin and eosin stain at 4X magnification and (B) 20X magnification.

## Ethical Considerations

Written informed consent for the publication of recognizable patient photographs and other identifiable material was obtained from the patient by the authors.

## Discussion

NTM comprise all mycobacterial species other than *Mycobacterium tuberculosis* and *Mycobacterium leprae* and are widely distributed in the environment. Cutaneous NTM infections most commonly occur following trauma or direct inoculation, including tattooing, and rapidly growing mycobacteria are most frequently implicated in tattoo-associated infections [8]. Clinical manifestations vary widely, from papules and pustules to nodules or abscesses, and lesions may appear weeks to years after tattooing [8]. Distinctly, our patient presented with an entirely asymptomatic, predominantly nonerythematous eruption.

Diagnosis of cutaneous NTM infection remains challenging due to variable clinical presentations, frequent absence of systemic symptoms, and nonspecific histopathologic findings. In this case, the asymptomatic nature of the eruption and the presence of a lichenoid vacuolar interface dermatitis, an atypical histologic pattern for infection, further complicated diagnosis, which could have been mistaken for an inflammatory tattoo reaction. Skin biopsy with mycobacterial culture remains the diagnostic gold standard, although prolonged incubation may delay treatment [9]. Emerging diagnostic techniques, including polymerase chain reaction assays, DNA probes, and matrix-assisted laser desorption ionization time-of-flight (MALDI-TOF) mass spectrometry can offer faster species identification once growth is achieved [9]. Nonetheless, antimicrobial susceptibility testing remains essential for therapy planning.

Management typically requires prolonged multidrug regimens, with treatment duration of 3‐12 months, depending on disease severity [6]. This case illustrates the critical transition from empiric short-course therapy to guideline-supported prolonged combination therapy, emphasizing the importance of early infectious disease involvement. From a public health perspective, tattoo-associated NTM infections raise concerns regarding ink contamination and the need for sterile tattooing practices. Limitations of this case include its single-patient design, inability to establish the source of NTM contamination, and limited long-term follow-up.

## Conclusion

This case describes an uncommon but increasingly seen presentation of tattoo-associated cutaneous NTM infection in an immunocompetent patient, characterized by asymptomatic multifocal lesions, atypical lichenoid vacuolar interface dermatitis, and delayed microbiologic diagnosis. Persistent, pigment-specific papules, particularly involving multiple tattoo sites or failing to respond to conventional therapy, should prompt consideration of NTM even in the absence of pain, erythema, or systemic symptoms. Early biopsy with mycobacterial culture and susceptibility testing can facilitate diagnosis and guide appropriate prolonged multidrug therapy. Recognizing these clinical clues and maintaining a low threshold for mycobacterial testing may help clinicians identify similar tattoo-associated infections earlier and avoid misdiagnosis as an inflammatory or allergic tattoo reaction.

## Abbreviations

*M abscessus*: *Mycobacterium abscessus*
MALDI-TOF: matrix-assisted laser desorption ionization time-of-flight
NTM: nontuberculous mycobacteria
